# Stool preparation under anaerobic conditions contributes to retention of obligate anaerobes: potential improvement for fecal microbiota transplantation

**DOI:** 10.1186/s12866-021-02325-9

**Published:** 2021-10-09

**Authors:** Hirotaka Shimizu, Katsuhiro Arai, Takashi Asahara, Takuya Takahashi, Hirokazu Tsuji, Satoshi Matsumoto, Ichiro Takeuchi, Reiko Kyodo, Yuichiro Yamashiro

**Affiliations:** 1 Division of Gastroenterology, Department of Medical Specialty, National Center for Child Health and Development, Tokyo, Japan; 2 Department of Pediatrics and Adolescent Medicine, Juntendo University Faculty of Medicine, Tokyo, Japan; 3 Yakult Central Institute, Tokyo, Japan; 4 Probiotics Research Laboratory, Juntendo University Graduate School of Medicine, Tokyo, Japan

**Keywords:** Obligate anaerobes, Fecal microbiota transplantation, Anaerobic preparation

## Abstract

**Background:**

Fecal microbiota transplantation (FMT) in patients with ulcerative colitis has shown variable efficacy depending on the protocol used. A previous randomized controlled trial reported that anaerobic preparation of donor stool contributes to improved efficacy. Despite the suggestion that viable obligate anaerobes would be decreased through aerobic handling, there have been only a limited number of reports on how these aerobic or anaerobic procedures affect the composition of viable microbiota in the fecal slurries used for FMT.

**Methods:**

We adopted 16S and 23S rRNA-targeted reverse transcription-quantitative polymerase chain reaction to quantify viable bacteria in fecal slurries. This study utilized specific primers designed to detect obligate anaerobes (including *Clostridium coccoides* group, *C. leptum* subgroup, *Bacteroides fragilis* group, *Bifidobacterium*, *Atopobium* cluster, and *Prevotella*) and facultative anaerobes (including total lactobacilli, *Enterobacteriaceae*, *Enterococcus*, *Streptococcus,* and *Staphylococcus*). We then calculated the ratio change (RC) between before and after mixing, and compared the resulting values between anaerobic-prep and aerobic-prep in samples fixed immediately after blending (RC_An0_ vs. RC_Ae0_) and in samples maintained (under anaerobic or aerobic conditions) for 1 h after blending (RC_An1_ vs. RC_Ae1_).

**Results:**

For most obligate anaerobes, the median RC tended to be less than 1, indicating that the number of obligate anaerobes was decreased by the blending procedure. However, in samples maintained for 1 h after blending, anaerobic-prep counteracted the decrease otherwise seen for the *C. coccoides* group and *B. fragilis* groups (*P* < 0.01 for both). The *C. leptum* subgroup also tended to show higher RC by anaerobic-prep than by aerobic-prep, although this effect was not statistically significant. Among facultative anaerobes, *Enterobacteriaceae*, *Enterococcus, and Staphylococcus* showed median RC values of more than 1, indicating that these organisms survived and even grew after mixing. Moreover, oxygen exposure had no significant influence on the survival of the facultative anaerobes.

**Conclusions:**

The conditions under which the blending procedure was performed affected the proportion of live anaerobes in fecal slurries. The obligate anaerobes tended to be decreased by blending processes, but anaerobic-prep significantly mitigated this effect. Anaerobic-prep may improve the efficacy of FMT by permitting the efficient transfer of obligate anaerobes to patients with ulcerative colitis.

**Supplementary Information:**

The online version contains supplementary material available at 10.1186/s12866-021-02325-9.

## Background

The advent of high-throughput DNA-based sequencing technology to classify bacteria according to 16S rRNA sequences has enabled identification of obligate anaerobes, a class that was difficult to capture by traditional culture techniques [1]. As a result, it was revealed that the obligate anaerobes comprise most of the healthy intestinal microbiota and have beneficial effects on our health [2]. For example, some obligate anaerobes (such as *Clostridium* cluster IV and XIVa) ferment dietary fibers and produce short-chain fatty acids, such as acetic acid and butyric acid [3]. Acetic acid has an antimicrobial effect on pathogenic bacteria [4], and butyric acid serves as a significant nutritional source for colonic epithelia and induces the differentiation of regulatory T cells [5].

Disruption of the bacterial balance is defined as dysbiosis. Recently, dysbiosis has been reported to be associated with the pathogenesis of various disorders, including inflammatory bowel disease, allergy, metabolic syndrome, and obesity. Indeed, in patients with ulcerative colitis (UC), a decrease in the abundance of obligate anaerobes (such as *Bacteroidetes* and *Firmicutes*) and an increase in abundance of facultative anaerobes (such as *Enterobacteriaceae* and *Enterococcaceae*) has been reported [6–8].

In 2013, Nood et al. reported the effect of fecal microbiota transplantation (FMT) for recurrent *Clostridioides* (formerly *Clostridium*) *difficile* infection (CDI), with an impressive cure rate of more than 90% [9]. Since then, FMT has become established as a therapy for recurrent CDI, and there is growing evidence for the utility of FMT against UC. In recent years, randomized controlled trials studying the application of FMT in patients with UC have reported varying results [10–13]. Unfortunately, the efficacy of FMT for the treatment of UC appears to be less than that of FMT for the treatment of CDI. Considering the decreases observed in beneficial obligate anaerobes in UC patients, delivery of these beneficial bacteria-rich fecal slurries might be the key to improving the efficacy of FMT for patients with UC.

However, in most studies, FMT suspensions were diluted with normal saline and homogenized using a blender in room air. Such oxygen exposure could cause the death of these beneficial bacteria because most are obligate anaerobes [14]. As a result, the number of live beneficial obligate anaerobes in fecal slurries may be decreased through the aerobic blending procedure. This compositional change of microbiota may be associated with the lower efficacy of FMT in patients with UC.

If anaerobic preparation, which reduces oxygen exposure, facilitates the retention of viable obligate anaerobes, outcomes of FMT might be improved. Indeed, Costello et al. observed the effectiveness of anaerobic preparation of donor stool in a randomized controlled trial of FMT for UC patients [13]. Moreover, Ding et al. reported a remarkably high response rate of step-up FMT strategy (clinical response rate at 1 month was as high as 74%), which utilizes an automatic system that can process fresh donor stool within 1 h of defecation [15]. These anaerobic or rapid preparation methods seem to reduce oxygen exposure and result in the survival of beneficial obligate anaerobes.

However, it has been difficult to determine how much the blending procedure impacts bacterial viability. Papanicolas et al. combined propidium monoazide (PMA) treatment with 16S rRNA gene amplicon sequencing to quantitatively assess the effect of the blending procedure on the abundance of viable bacteria in fecal slurries [16]. Those authors processed samples with PMA within 15 min of blending and reported that aerobic blending resulted in significantly lower bacterial viability (19%) than did anaerobic blending (50%). Papanicolas et al. further showed that butyrate and acetate production was decreased in aerobically prepared samples compared to production in anaerobically processed samples. These findings illustrated the importance of anaerobic stool processing.

**Fig. 1:**
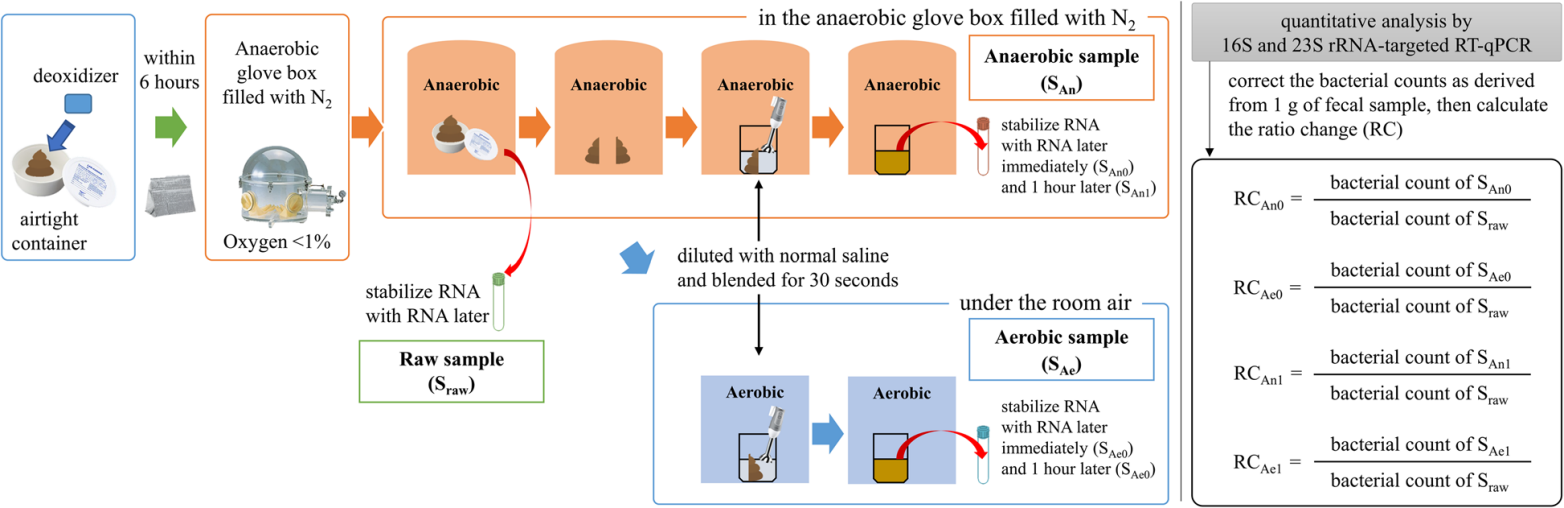
Stool collection and anaerobic and aerobic preparation. Fecal samples from 16 healthy adults were transferred under anaerobic conditions using an airtight container and deoxidizer and aliquoted into two portions in an anaerobic glove box. Each aliquot then was diluted with normal saline and blended for 30 s, either in the anaerobic glove box (anaerobic-prep) or under room air (aerobic-prep). Fecal samples were collected before blending (S_raw_), and then immediately (0 h) and 1 h after anaerobic-prep (S_An0_ and S_An1_, respectively), or at 0 and 1 h after aerobic-prep (S_Ae0_ and S_Ae1_, respectively)

In the present study, we collected 1-h samples that were stabilized by RNAlater after 1-h maintenance under the anaerobic or aerobic conditions, in addition to 0-h samples that were stabilized immediately after blending (Fig. 1). We considered that the 1-h samples reflected the actual clinical settings in which FMT typically would be performed. Because the administration of FMT solution by endoscope or enema tube usually takes time, the timely insertion of FMT solution within 15 min seems to be difficult.

To evaluate bacterial viability, we adopted the Yakult Intestinal Flora-SCAN (YIF-SCAN®) system. This system utilizes reverse transcription-quantitative polymerase chain reaction (RT-qPCR) to target bacterial rRNA. Since rRNA in dead cells is rapidly degraded, this method permits quantitative determination of the number of viable cells. Moreover, the sensitivity of this system is theoretically 100–1000 fold higher than that of qPCR assays that target rRNA genes (which exist at 5–10 copies per bacterial cell), as the rRNA molecules exist abundantly (approximately 10^4^ copies per bacterial cell) [14, 17]. Therefore, RT-qPCR is a more sensitive assay and is expected to detect rRNA only from live bacteria in the specimens [17].

Using this technique, we sought to examine the effectiveness of anaerobic preparation in preserving viable obligate anaerobes in real-world clinical settings.

## Results

### Fecal bacterial composition in samples before blending (S_raw_)

**Fig. 2:**
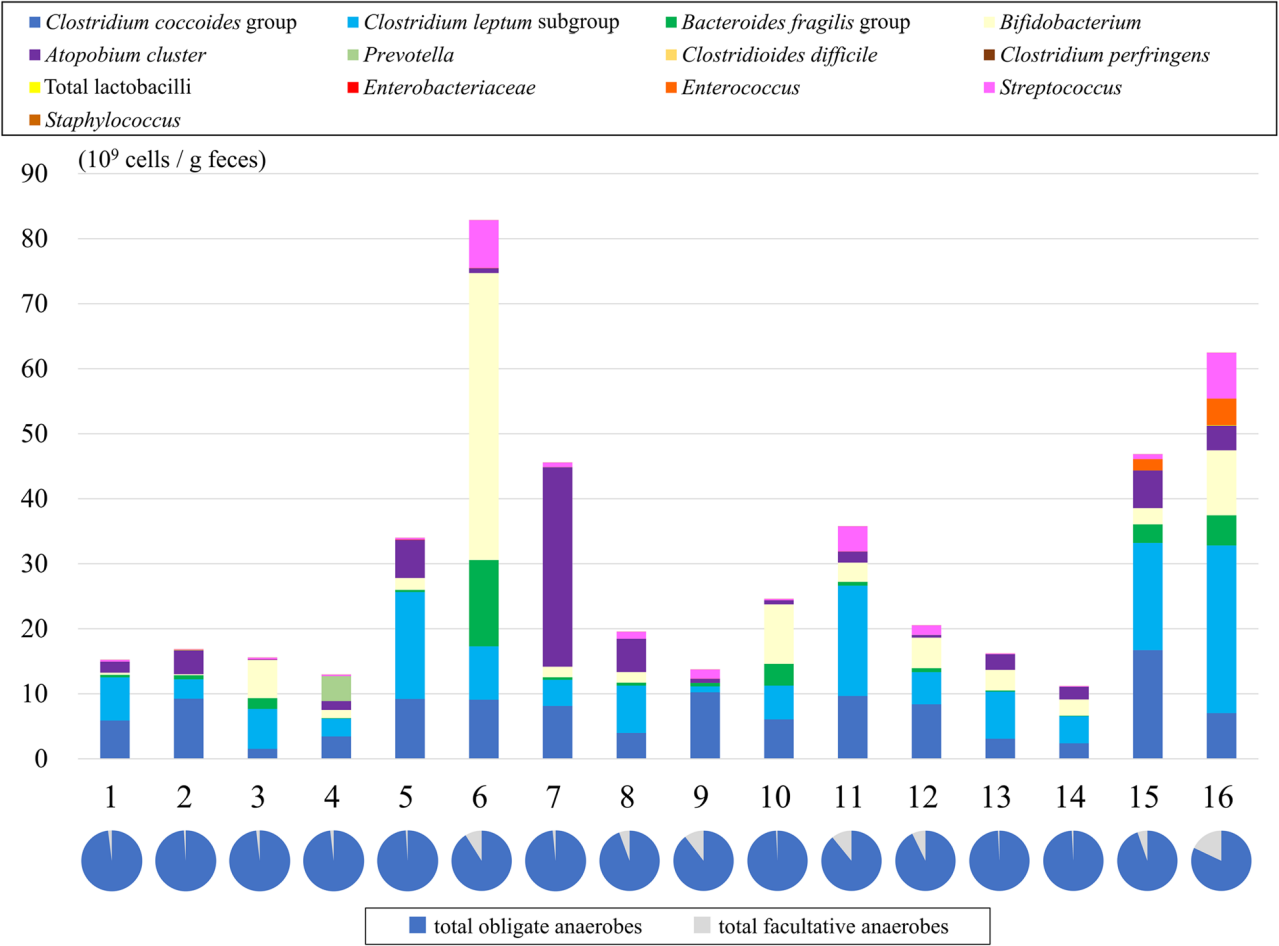
The bacterial composition of raw samples from 16 subjects. Each stacked bar represents bacterial counts per 1 g of feces, as determined by 16S and 23S quantitative reverse transcription-polymerase chain reaction (RT-qPCR). In all subjects (except subjects 6 and 7), obligate anaerobes, including the *C. coccoides* group and *C. leptum* subgroup, accounted for the majority of the fecal microbiota. The proportion of the facultative anaerobes was significantly lower than that of the obligate anaerobes

The bacterial composition of raw samples (before blending of samples) from the 16 subjects is shown in Supplementary Table 1 (where data are shown in logarithmic form). The stacked bar chart is shown in Fig. 2 (where data are shown in real numbers).

The proportion of the facultative anaerobes was significantly lower than that of the obligate anaerobes. In most samples (except for subjects 6 and 7), the *Clostridium coccoides* group (corresponding to *Clostridium* cluster XIVa) and the *C. leptum* subgroup (corresponding to *Clostridium* cluster IV) accounted for the majority of the fecal microbiota. Subject 6 was rich in the *Bifidobacterium* and *Bacteroides fragilis* groups, and subject 7 was abundant in the *Atopobium* cluster.

### Comparison of the ratio change in viable bacteria before and after preparation

In the present study, we calculated the ratio change (RC) in the number of viable bacteria before and after the preparation. For the bacterial groups whose numbers were below the detection limit, the value was set to 50% of each lower limit. The RC was then calculated by dividing the number of bacteria after mixing (S_An0_, S_An1_, S_Ae0_, or S_Ae1_) by the original number of bacteria (S_raw_). The formula for the RC is shown in Fig. 1. We then compared bacterial viability between anaerobic-prep and aerobic-prep.

**Fig. 3:**
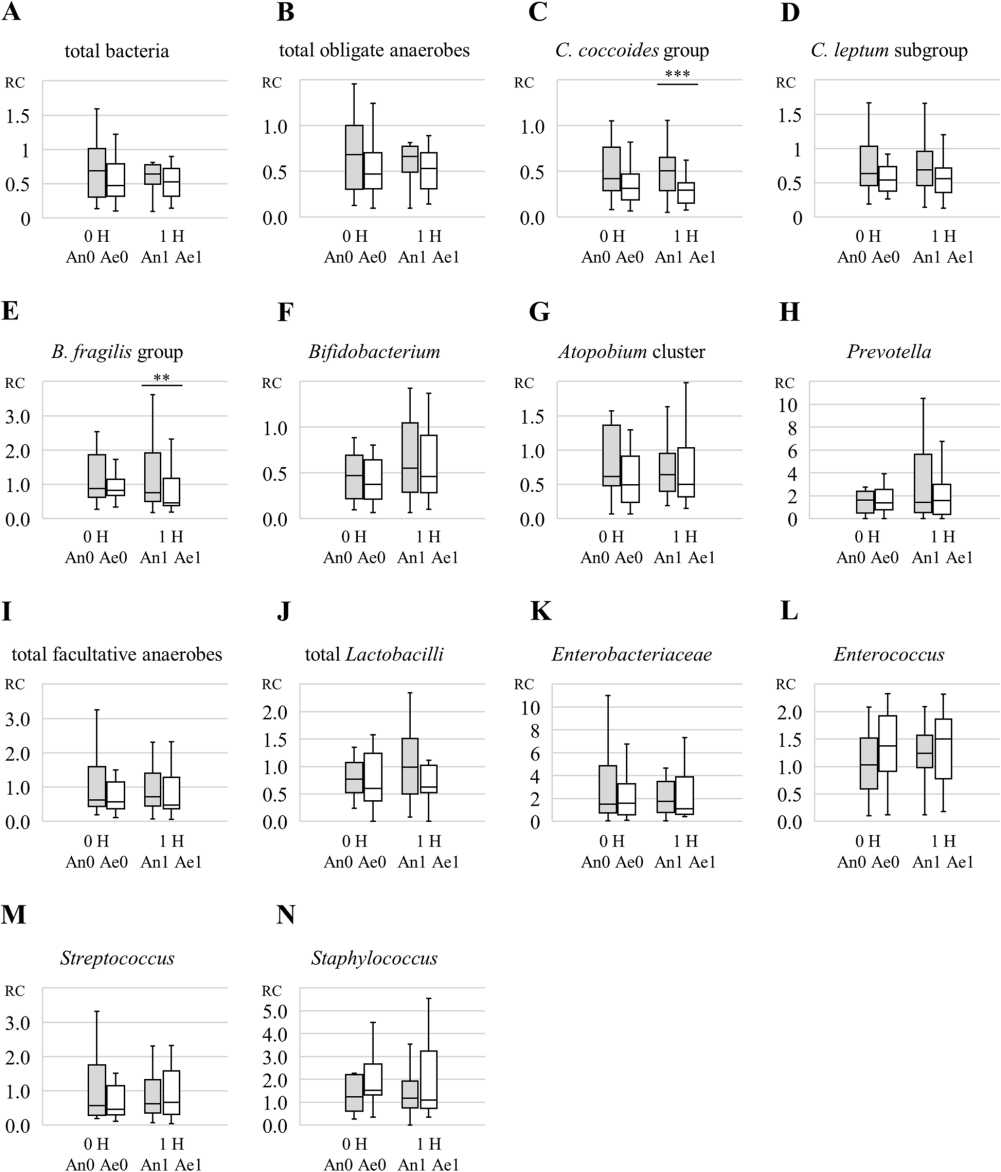
Comparison of the ratio change in the obligate and facultative anaerobes. These charts show the comparison of the ratio change (RC) in the number of live bacteria for **A:** total bacteria, **B:** total obligate anaerobes, **C–H:** each of the obligate anaerobes, **I:** total facultative anaerobes, and **J–N:** each of the facultative anaerobes. An RC value of more than 1 indicates that the bacterial group survived and even grew after the blending procedure. The box plot depicts the median and interquartile range and error bars represent the minimum and maximum values of the RC from 16 healthy volunteers. Each box plot compared RCs of anaerobic-prep and aerobic-prep, for samples stabilized immediately after blending (RC_An0_ and RC_Ae0_), and also for samples stabilized after maintenance for 1 h under the respective anaerobic or aerobic conditions (RC_An1_ and RC_Ae1_). Significant differences are indicated by asterisks (**P < 0.01, ***P < 0.001; Wilcoxon signed-rank sum test)

Fig. 3 shows the comparison of RCs in the number of live bacteria for **A**: total bacteria, **B**: total obligate anaerobes, **C–H**: each of the obligate anaerobes, **I**: total facultative anaerobes, and **J–N**: each of the facultative anaerobes. Each box plot compared the RCs of anaerobic-prep and aerobic-prep in samples stabilized immediately after blending (RC_An0_ and RC_Ae0_), and also in samples stabilized following 1 h of maintenance under the respective anaerobic or aerobic conditions (RC_An1_ and RC_Ae1_).

In samples stabilized immediately after blending (0-h samples; RC_An0_ and RC_Ae0_), the median RC values of the obligate anaerobes were less than 1 (Figs. 3 A–H). The RC of anaerobic-prep (RC_An0_) tended to be higher than that of the aerobic-prep (RC_Ae0_), although the differences did not reach statistical significance.

For samples stabilized at 1 h after blending (1-h samples), the RC_An1_ was significantly higher than the RC_Ae1_ for the *C. coccoides* group (Fig. 3C) and *B. fragilis* group (Fig. 3E) (*P* < 0.001 and *P* < 0.01, respectively). Additionally, the RC_An1_ for the *C. leptum* subgroup tended to be higher than the RC_Ae1_ (Fig. 3D), although this difference was not statistically significant. There were no significant differences between the RC_An1_ and RC_Ae1_ values for the other obligate anaerobes (*Bifidobacterium* cluster, *Atopobium* cluster, and *Prevotella*).

For facultative anaerobes, the median RC values for the *Enterobacteriaceae* (Fig. 3K), the *Enterococcus* (Fig. 3L), and the *Staphylococcus* (Fig. 3N) tended to be more than 1, indicating that these organisms survived and even grew after the mixing procedure. For *Enterobacteriaceae*, *Enterococcus*, *Streptococcus*, and *Staphylococcus,* there was no significant difference between RC_An1_ and RC_Ae1_. These results indicated that oxygen exposure had no significant influence on the survival of facultative anaerobes.

## Discussion

This study compared the number of live bacteria in fecal slurries for FMT between anaerobic-prep and aerobic-prep using RT-qPCR targeting bacterial rRNA. Notably, a larger number of viable obligate anaerobes were retained in slurries processed by anaerobic-prep.

In a previous randomized controlled trial, Costello et al. [13] assessed changes in fecal microbiota by 16S rRNA gene sequencing. Those authors reported that increases in the abundances of *Anaerofilum pentosovorans*, *Bacteroides coprophilus*, and *Clostridium methylpentosum* after FMT were strongly associated with the improvement of UC. Costello et al. also noted that organisms whose abundances were positively related to improved outcomes were primarily obligate anaerobes. In the present study, we conducted RT-qPCR targeting bacterial rRNA to quantify live bacteria. In this taxonomy, the *C. coccoides* group (*Clostridium* cluster XIVa) consists of the genera *Ruminococcus, Eubacteria*, *Coprococcus*, and *Lachnospira.* Likewise, the *C. leptum* subgroup (*Clostridium* cluster IV) contains the genera *Anaerofilum, Faecalibacterium*, and *Ruminococcus,* and the *B. fragilis* group includes the major *Bacteroides* species. Notably, most bacteria associated with treatment response in the above RCT belonged to one of these three groups. In this regard, it is significant that anaerobic-prep was related to retention of these three groups of bacteria.

In the current work, the abundance of facultative anaerobes, such as *Enterobacteriaceae* and *Enterococcus,* tended to increase after processing. The increased abundance of these facultative anaerobes is not favorable because these anaerobes have been reported to increase in active-phase UC patients. However, the absolute number of facultative anaerobes remained markedly lower than that of the obligate anaerobes (Table 1). Conversely, for obligate anaerobes, the RCs obtained by anaerobic-prep and aerobic-prep differed by 0.3 or less. However, these RC differences would significantly impact the absolute number of obligate anaerobes, given that these organisms are present at billions per gram of feces at baseline.

**Table 1:** Fecal counts of gut microbiota as determined by RT-qPCR

	0H samples			1H samples		
	Anaerobic samples	Aerobic samples	*P*	Anaerobic samples	Aerobic samples	*P*
	(n = 16)	(*n* = 16)		(n = 16)	(n = 16)	
Total bacterial counts	10.1 ± 0.3	10.0 ± 0.3		10.2 ± 0.3	10.1 ± 0.3	
Obligate anaerobe						
*Clostridium coccoides* group	9.4 ± 0.4	9.2 ± 0.4		9.4 ± 0.4	9.2 ± 0.4	**0.0042
*C.leptum* subgroup	9.6 ± 0.4	9.5 ± 0.4		9.6 ± 0.4	9.5 ± 0.4	**0.1750
*Bacteroides fragilis* group	8.8 ± 0.6	8.8 ± 0.6		8.8 ± 0.6	8.6 ± 0.6	
*Bifidobacterium*	8.9 ± 0.7	8.8 ± 0.8		9.0 ± 0.7	8.9 ± 0.7	
*Atopobium* cluster	9.1 ± 0.5	9.0 ± 0.4		9.1 ± 0.5	9.0 ± 0.5	
*Prevotella*	5.9 ± 1.9	6.1 ± 1.4		5.9 ± 1.9	5.6 ± 2.0	
*C.difficile*	1.2 ± 0.0	1.3 ± 0.4		1.4 ± 0.9	1.2 ± 0.0	
*C.Perfringens*	1.5 ± 1.0	1.4 ± 0.8		1.2 ± 0.0	1.8 ± 1.3	
Faculative anaerobe						
Total lactobacilli	5.8 ± 1.0	5.7 ± 1.4		6.0 ± 1.2	5.6 ± 1.5	
*Lactobacillus* (formerly *L. gasseri* subgroup)	4.2 ± 2.1	4.3 ± 2.0		4.4 ± 2.2	4.2 ± 2.1	
*Levilactobacillus brevis* (formerly *L. brevis*)	1.8 ± 1.3	1.6 ± 1.2		1.6 ± 1.1	1.6 ± 1.2	
*Lacticaseibacillus* (formerly *L. casei* subgroup)	2.7 ± 1.8	3.7 ± 1.7		3.4 ± 1.9	3.4 ± 1.9	
*Limosilactobacillus fermentum* (formerly *L. fermentum*)	3.0 ± 1.7	3.2 ± 1.6		2.9 ± 1.6	3.0 ± 1.5	
*Fructilactobacillus fructivorans* (formerly *L. fructivorans*)	1.2 ± 0.0	1.2 ± 0.0		1.2 ± 0.0	1.2 ± 0.0	
*Lactiplantibacillus* (formerly *L. plantarum* subgroup)	3.4 ± 1.7	3.4 ± 1.7		3.8 ± 1.3	3.4 ± 1.7	
*Limosilactobacillus* (except *L. fermentum*) (formerly *L. reuteri* subgroup)	3.4 ± 1.9	3.2 ± 1.8	*0.0415	3.2 ± 2.0	3.3 ± 1.9	
*Ligilactobacillus and Liquorilactobacillus* (formerly *L. ruminis* subgroup)	2.5 ± 2.2	2.3 ± 2.0		2.5 ± 2.1	2.6 ± 2.1	
*Latilactobacillus* (formerly *L. sakei* subgroup)	2.4 ± 1.9	2.4 ± 1.9		2.6 ± 2.0	2.4 ± 2.0	
*Enterobacteriaceae*	6.5 ± 0.7	6.4 ± 0.6		6.5 ± 0.7	6.4 ± 0.6	
*Enterococcus*	5.6 ± 2.1	5.7 ± 2.1		5.9 ± 1.8	5.7 ± 2.1	
*Streptococcus*	8.7 ± 0.6	8.6 ± 0.6		8.6 ± 0.6	8.5 ± 0.6	
*Staphylococcus*	5.1 ± 0.9	5.2 ± 0.7		4.9 ± 1.1	5.1 ± 0.7	

The results are displayed as the means ± SD (log10 cells/g of feces)

* *p* < 0.05, ** *p* < 0.01

As mentioned previously, Papanicolas et al. reported that anaerobic-prep was associated with improved viability of anaerobes, but noted that only 50% of the bacterial content was viable even with anaerobic-prep. Those data were obtained based on samples treated with PMA within 15 min of blending. Our results, obtained from samples treated immediately after blending (RC_An0_), are consistent with those of Papanicolas et al. We also showed that samples maintained for 1 h under anaerobic conditions (RC_An1_) exhibited viabilities essentially identical to those collected and stabilized immediately after processing (RC_An0_). We believe that the sampling in our study better reflects the real-world setting, given that the fecal slurries employed for FMT seldom are transplanted to patients within 15 min of collection. Indeed, the interval from blending to administration would typically exceed 15 min, considering the time required for the insertion of a colonoscope or enema tube.

As mentioned above, this study adopted the YIF-SCAN® system for the analysis of fecal bacteria. This system utilizes RT-qPCR targeting bacterial rRNA as an alternative to DNA molecules and enables live gut bacteria to be quantified [17, 18]. Unlike RT-qPCR targeting RNA and culture counts, qPCR targeting rRNA genes and 4′,6-diamidino-2-phenylindole (DAPI) staining counts are relatively constant, even as bacterial cells die. This consistency reflects the fact that qPCR and DAPI staining quantify DNA molecules derived from viable cells as well as those from dead cells; DNA molecules might not degrade rapidly in the dead cells during the late period of culturing [19, 20]. The YIF-SCAN® system has already been validated for its ability to quantify major human gut bacteria accurately and sensitively [21, 22].

There are several limitations associated with this study. First, the sample size of this study was relatively small. However, significantly higher RCs for the *C. coccoides* group and *B. fragilis* group, and a tendency toward higher RC for the *C. leptum* subgroup, were observed following anaerobic-prep, even with such a small sample size. Overall, anaerobic-prep is considered to be effective in maintaining obligate anaerobes in suspensions intended for use in FMT. Second, this study focused solely on the ratio change in the number of bacteria. We did not assess metabolomic functions, including the microbial capacity for producing butyrate and acetate. Third, our findings demonstrated the need to compare both anaerobic-prep and aerobic-prep in clinical trials to clarify whether these preparation methods have any impact on the effectiveness of FMT for UC patients. In this regard, further investigation is needed.

## Conclusion

In conclusion, anaerobic preparation of fecal slurries contributed to the retention of obligate anaerobes, even in samples maintained under anaerobic conditions for 1 h post-blending, compared with the conventional aerobic processing. Our findings suggest the importance of anaerobic handling of the donor stool during transportation, mixing, and preservation. These anaerobic preparations might improve the outcome of FMT for UC patients by facilitating the transfer of viable obligate anaerobes.

## Methods

### Collection and processing of fecal samples

We collected fecal samples from each of 16 healthy Japanese volunteers; each volunteer provided informed consent prior to sample collection. Figure 1 shows the stool preparation process. To minimize oxygen exposure during transportation, fecal samples were transferred under anaerobic conditions using an airtight container equipped with a deoxidizer (Anaeropack, SUGIYAMA-GEN Co., Ltd., Tokyo, Japan). Fecal samples were placed inside an anaerobic glove box (AS ONE Corporation, Osaka, Japan) within 6 h of passage. In the glove box, the oxygen concentration was maintained at < 1% by replacing air with nitrogen using a reverse air pump. As baseline samples, fecal samples were collected and stabilized with RNAlater (Ambion, Austin, TX, USA) before blending (S_raw_). Fecal samples then were aliquoted into two portions by handling within the glove box. Each aliquot was blended with normal saline water for 30 s, either in the anaerobic glove box (anaerobic-prep) or under room air (aerobic-prep).

As shown in Fig. 1, samples S_An0_ and S_Ae0_ were collected and fixed with RNAlater immediately after blending under anaerobic or aerobic conditions, respectively.

In addition to 0-h samples, we also collected 1-h samples that were stabilized after being maintained for 1 h under the anaerobic or aerobic conditions. These 1-h samples were designated S_An1_ and S_Ae1_, respectively.

To stabilize RNA, each fecal sample (S_raw_, S_An0_, S_An1_, S_Ae0_, and S_Ae1_) was placed into a tube containing 2 mL of RNAlater. These samples then were stored at 4 °C pending analysis of the fecal microbiota.

### Analysis of live fecal bacteria

For the analysis of fecal bacteria, we adopted the YIF-SCAN® system, which utilizes RT-qPCR targeting 16S and 23S rRNA. Using previously described methods [21–25], we extracted total RNA fractions to quantify the bacteria present in the samples. The specificity of the RT-qPCR assay using group-, genus-, or species-specific primers also was determined as described previously [21–25]. Three serial dilutions of the extracted RNA samples were used for bacterial rRNA-targeted RT-qPCR. Threshold cycle values in the linear range of the assay were applied to the standard curve. These data were used to calculate the number of viable bacteria in each sample. For all samples, bacterial PCR products were normalized to the content of a 1-g fecal sample.

### Statistical analysis

All statistical analyses were performed using EZR version 1.38 (Saitama Medical Center, Jichi Medical University, Saitama, Japan), a graphical user interface for R version 3.5.2 (The R Foundation for Statistical Computing, Vienna, Austria) [26]. Data are expressed as the median and interquartile range for data with skewed distribution. The Wilcoxon signed-rank sum test was used for data analysis, and *P* < 0.05 was considered statistically significant.

## Supplementary Information

**Additional file 1.**

## Data Availability

The datasets generated and analyzed during the current study are available in the figshare repository. The raw data of the bacterial counts and 16S or 23S rRNA gene-targeted primers used in this study have been deposited at figshare repository, with persistent web link: 10.6084/m9.figshare.14229662
